# Synchronous Colon Adenocarcinoma With Right Inguinal Lymph Node Metastasis: A Case Report and Brief Literature Review

**DOI:** 10.7759/cureus.11501

**Published:** 2020-11-16

**Authors:** Ahmed M Alzahrani, Abdulaziz Alshehri

**Affiliations:** 1 Surgery, Majmaah University, Majmaah, SAU; 2 General Surgery, King Abdulaziz Medical City, Jeddah, SAU

**Keywords:** inguinal lymph nodes, metastasis, colon cancer

## Abstract

Colon cancer is one of the common cancers and a major reason for cancer-related deaths with reported metastasis to the lungs and liver. Metastasis to inguinal lymph nodes from the adenocarcinoma of the colon is very rare and needs to be managed distinctly. We present a case of moderately differentiated adenocarcinoma of colon confirmed pathologically in an elderly male patient with locally extensive malignancy with involvement of the right-sided inguinal lymph nodes.

## Introduction

Colon cancer is well known for its metastatic capacity [1]. In most cases, it metastasizes to the liver and lungs if not caught early and treated properly. This occurrence is supported by its ability to metastasize through the hematogenous and lymphatic spread. However, it is extremely rare to encounter metastasis to inguinal lymph nodes from the colonic origin. On the contrary, lower rectal cancer has shown its ability to metastasize to inguinal lymph nodes, though it is not frequently encountered.

To our knowledge, there are six case reports of colon cancer with inguinal lymph nodes metastases [2-5], although certain characteristics might vary as to the location and means of spread. We report our case of a patient presenting with a synchronous colon adenocarcinoma with right inguinal lymph node metastasis.

## Case presentation

**Figure 1:**
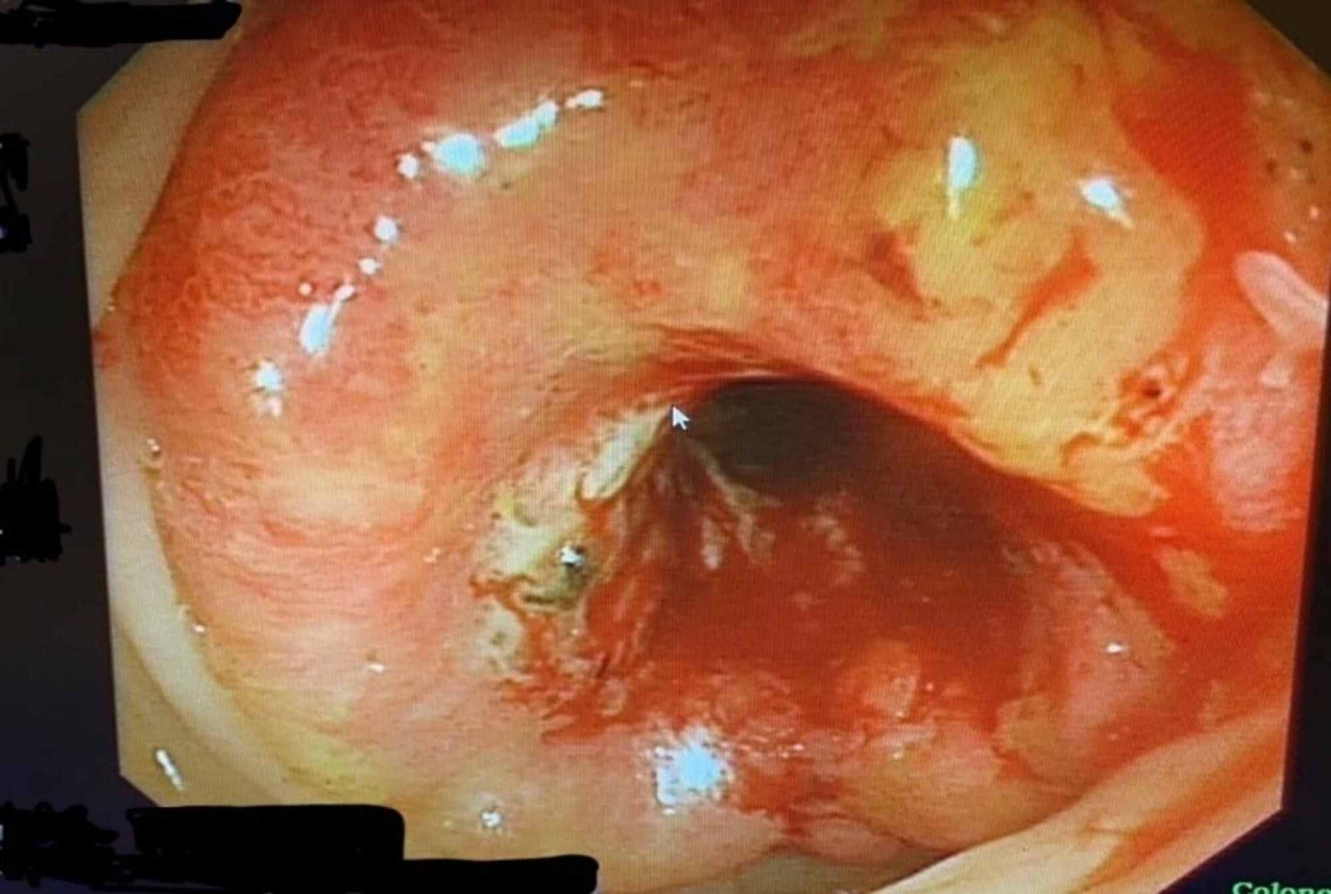
A colonoscopy view shows annular mass in descending colon.

**Figure 2:**
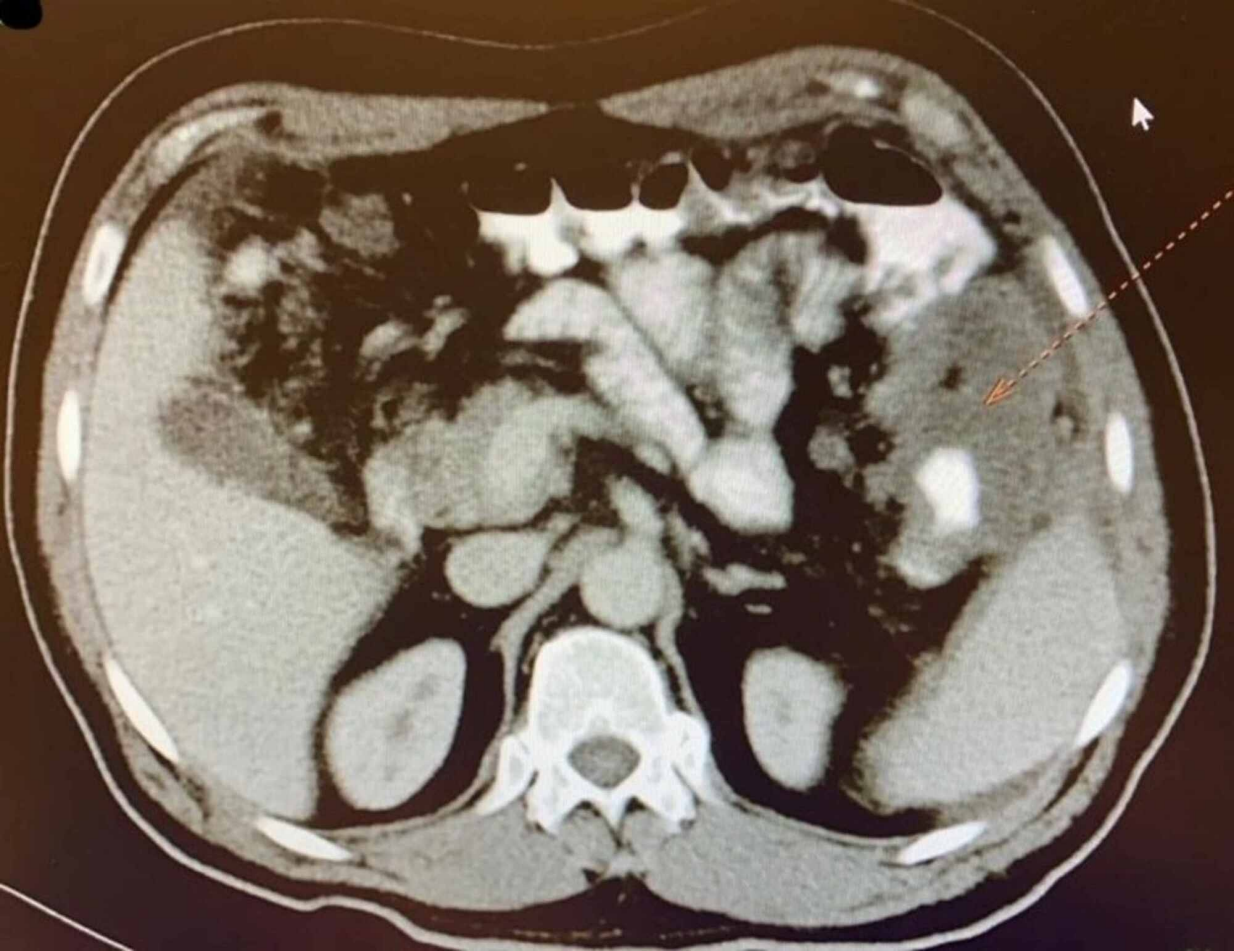
A cross-sectional slice of CT abdomen showing left colonic mass with extramural extension.

**Figure 3:**
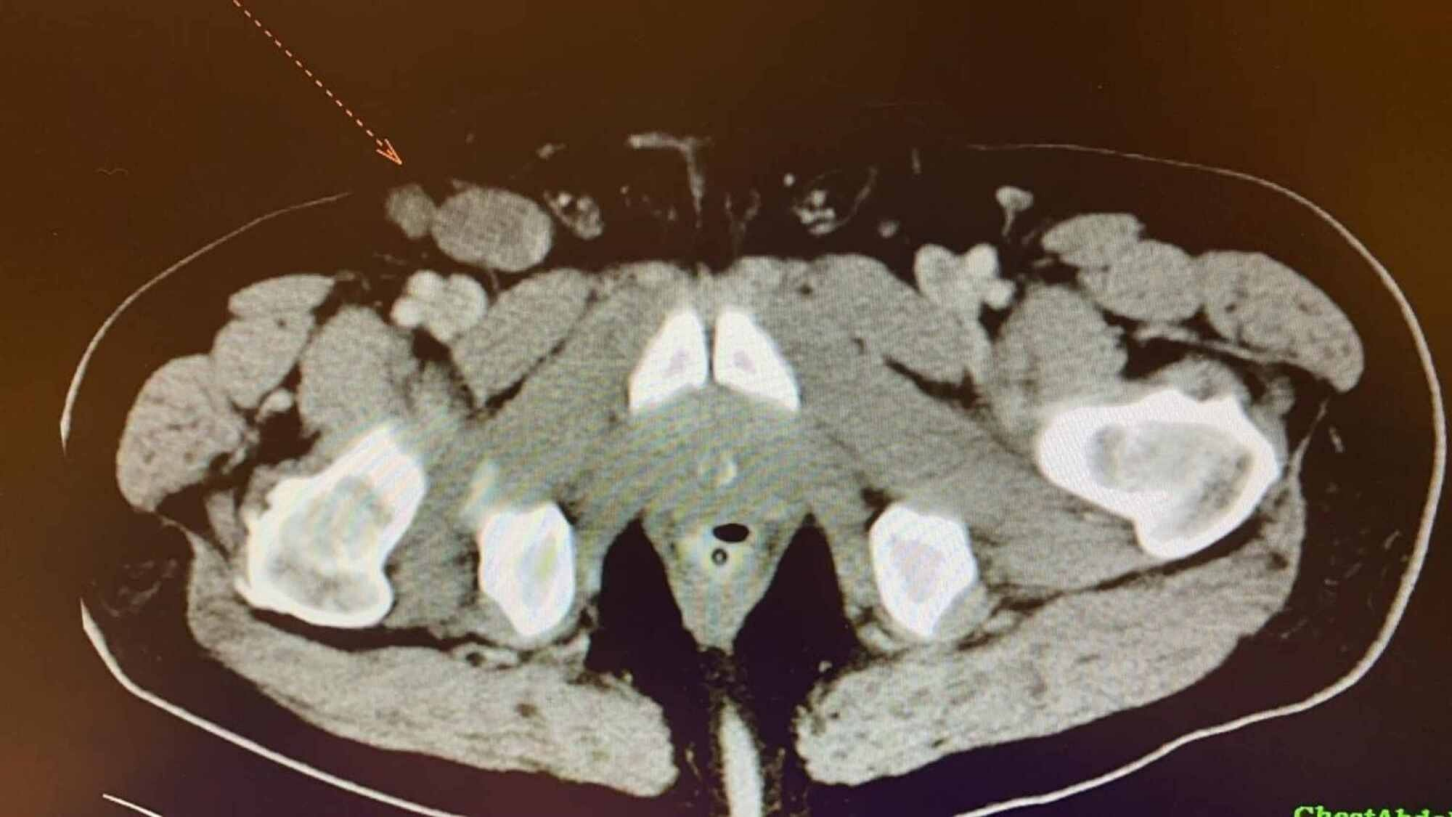
A cross-sectional slice of CT abdomen showing enlarged right inguinal lymph node.

A 59-year-old male presented to the family practitioner complaining of melena, abdominal discomfort, weight loss, loss of appetite for one month, and right inguinal swelling. Then, he was referred to the gastroenterologist for a colonoscopy which showed a splenic flexure annular mass (Figure 1) 80 cm away from the anal verge that was biopsied. The pathology examination revealed moderately differentiated adenocarcinoma. The patient then underwent a staging workup which included chest, abdomen, and pelvis CT imaging. It revealed the presence of locally extensive disease (Figure 2) that included multiple splenic focal lesions, multiple peritoneal deposits, and a large right-sided inguinal lymph node (Figure 3). No liver or pulmonary metastases were detected.

After complete workup, the patient was referred to our service to perform an excisional biopsy of the right inguinal lymph node. Excision was done and the pathology report revealed the presence of metastatic moderately differentiated mucinous adenocarcinoma of colorectal origin. No further surgical intervention was offered to the patient as the multidisciplinary team decision of the Tumor Board labeled him as a metastatic disease that warrants palliative chemotherapy.

## Discussion

Retrospective studies and the Saudi Cancer registry noted an increase in colon cancer in Saudi Arabia over the past decade and distant metastasis was diagnosed in about 28.4% of patients at the presentation of which rectal cancers account for 41% [1]. Colorectal carcinoma usually metastasizes to the liver and lungs, but there are few cases reported of spread to rare sites like the heart, skeletal muscle, spleen, ovary, and others [6-10]. It is rare for colon cancer to metastasize to inguinal lymph nodes, as anatomically the venous and lymphatic drainage does not drain towards the external iliac system.

 Our case is of a patient with locally advanced disease with a right inguinal lymph node metastases. It is considered to be an extremely rare incidence [2-5]. The means by which colon cancer spreads to inguinal lymph nodes remains to be uncertain. Most of the aforementioned case reports hypothesized that the method of spread is attributed to direct abdominal wall cancerous invasion, howbeit the remaining case reports did not demonstrate abdominal wall invasion. This fact attenuates this hypothesis. Kitano et al. demonstrated that the primary tumor invaded the abdominal wall neither macroscopically nor microscopically. More importantly, they have displayed the absence of regional lymphadenopathy [2]. In addition, Uehara et al. described this phenomenon by depicting how direct abdominal wall invasion can result in external iliac artery lymphadenopathy through the transmission of cancerous cells through the inferior epigastric artery lymphatic channels [3]. On the other hand, Tanabe et al. showed metachronous transmission to the inguinal lymph nodes associated with abdominal wall metastases two years following the conclusion of treatment [4]. Metastasis from both right and sigmoid colon has been noticed [2-5]. A case reported by Hakeem et al. noted the cecal carcinoma spreading to contralateral inguinal nodes [11].

 However, ours seems to be the first case to originate from the splenic flexure. We believe that the hypothesis of direct abdominal wall invasion is true to our case due to the fact of the presence of peritoneal deposits.

## Conclusions

We have encountered an extremely rare case that constitutes a challenge to the treating surgeon. A case of locally advanced colonic adenocarcinoma with peritoneal deposits and right inguinal lymphadenopathy. Further evidence is of utmost significance in order to support a certain plan of care since the management of such conditions varied drastically between different case reports.
